# Acute myeloid leukemia – strategies and challenges for targeting oncogenic Hedgehog/GLI signaling

**DOI:** 10.1186/s12964-017-0163-4

**Published:** 2017-01-25

**Authors:** Fritz Aberger, Evelyn Hutterer, Christina Sternberg, Pedro J. del Burgo, Tanja N. Hartmann

**Affiliations:** 1 Cancer Cluster Salzburg, Department of Molecular Biology, Paris-Lodron University of Salzburg, Hellbrunner Strasse 34, 5020 Salzburg, Austria; 2 Cancer Cluster Salzburg, Salzburg Cancer Research Institute (SCRI) - Laboratory for Immunological and Molecular Cancer Research (LIMCR), 5020 Salzburg, Austria; 3 Third Medical Department with Hematology, Medical Oncology, Hemostaseology, Infectious Disease and Rheumatology, Oncologic Center, Paracelsus Medical University Salzburg, Muellner Hauptstrasse 48, 5020 Salzburg, Austria

**Keywords:** Acute myeloid leukemia, Cancer stem cells, Hedgehog (HH) signaling, GLI proteins, Non-canonical Hedgehog/GLI signaling, Combination therapy

## Abstract

Treatment of acute myeloid leukemia (AML), an aggressive and heterogeneous hematological malignancy, remains a challenge. Despite advances in our understanding of the complex genetics and biology of AML pathophysiology, these findings have been translated to the clinic with only limited success, and poor outcomes persist for the majority of patients. Thus, novel treatment strategies are clearly needed for achieving deeper and prolonged remissions and for avoiding the development of resistance. Due to its profound role in (cancer) stem cell biology and differentiation, the Hedgehog (HH)/Glioma-associated Oncogene Homolog (GLI) signaling pathway may be an attractive novel therapeutic target in AML. In this review, we aim to provide a critical and concise overview of the currently known potential and challenges of HH/GLI targeting. We describe the biological role of the HH/GLI pathway in AML pathophysiology. We specifically focus on ways of targeting non-canonical HH/GLI signaling in AML, particularly in combination with standard treatment regimens, which may overcome some hurdles observed with approved HH pathway inhibitors in solid tumors.

## Background

Acute myeloid leukemia (AML) is an aggressive hematological neoplasm with a highly diverse clinical course. Known prognostic factors include age, complex karyotype, mutations, presence of elevated white blood cell counts, antecedent hematologic disease, and prior chemo/radiotherapy for another malignancy [1]. With the advent of newer technologies such as next generation sequencing, the prognostic relevance of specific mutations and karyotypes is becoming more and more recognized and is reflected in the current revision of the AML classification of the World Health Organization (WHO) [2]. The 2016 revision updates the prior classification in an attempt to incorporate new clinical, morphological, immunophenotypic, cytogenetic and molecular genetic markers that have emerged in recent years. In contrast, in the older French-American-British (FAB) criteria, the classification of AML is solely based upon morphology, i.e. the degree of cell differentiation and maturation [3]. Moreover, the FAB classification used a cut-off of ≥ 30% blasts in the blood or bone marrow (BM) for AML diagnosis, which was adapted by the WHO to ≥ 20% since several studies reported a similar prognosis in terms of survival for patients with 20–29% blasts as for those with ≥ 30% blasts in the BM [4–8].

In order to be successful, AML treatment mainly requires management of the BM and systemic disease. Therefore, AML therapy is based on systemic combination chemotherapy and usually includes two treatment phases: firstly, the achievement of remission (induction) and secondly, the consolidation of remission. Current treatment for previously untreated AML in fit/younger patients is composed of two therapeutics, cytarabine (Ara-C) and an anthracycline such as daunorubicin (“7 + 3 induction therapy”), with a complete response/remission (CR) rate of about 65% [9]. This can optionally be accompanied by thioguanine [10], although due to only little available data it is not possible to infer a superiority of this extended combination. Another optional addition is etoposide [11], which might prolong the duration of the initial response. Moreover, different forms and doses of anthracycline can influence the treatment outcome, so it was found that in younger patients idarubicin is more effective than daunorubicin, however, the doses tested have not been the same [9, 12–14] and no significant survival benefit was found [15].

A randomized study by the Eastern Cooperative Oncology Group (ECOG) showed that after only a short term CR all patients without consolidation therapy relapsed [16] and only few successful chemotherapies without relapse after a single treatment cycle have been reported [17]. Therefore, a consolidation therapy after initial remission is mandatory in order to achieve a curative effect. The current efforts in consolidation therapy comprise relatively short and intense or higher doses of chemotherapy with regimens also used in initial treatment as well as autologous or allogeneic hematopoietic stem cell (HSC) transplantation after BM chemoradiation/ablative therapy [18].

Older or unfit patients, however, often do not endure a high dose remission initiating treatment. Thus, those patients benefit more from receiving low-dose Ara-C [19] or hypomethylating agents like decitabine and azacitidine (5-Aza), which was initially approved for treatment of myelodysplastic syndrome (MDS) [20, 21]. Preliminary data from a recent phase III trial showed comparable overall survival for patients older than 65 years receiving either 5-Aza, conventional therapy of best supportive care or the 7 + 3 induction therapy [22].

Despite achievement of CR after initiation/consolidation therapy the majority of AML patients eventually relapse, either due to a lack of response or the development of drug resistance. Thus, relapsed/refractory AML (rrAML) is rather common and unfortunately very difficult to manage due to limited availability of effective therapies [23]. Currently, treatment of rrAML, depending on the patient’s fitness, includes non-/intensive chemotherapy regimens and/or HSC transplantation as well as a combination of investigational agents and high dose Ara-C, often also the enrollment in clinical trials [24]. However, standard AML treatment has not seen many modifications within the last decades and new therapeutic approaches are needed, especially for unfit patients and those with negative prognostic factors, which is highly challenging considering the heterogeneity of the various prognostic and molecular AML subgroups. This need is reflected in the numerous new treatment options presently under development and in clinical trials including combination approaches, novel formulations of cytotoxic chemotherapy and hypomethylating agents as well as other epigenetic modifiers, antibody-drug conjugates and molecularly targeted agents like cell cycle and signaling inhibitors [25–27].

Moreover, since there is increasing evidence that levels of minimal residual disease (MRD) after induction therapy are a relevant risk factor, the monitoring of MRD during remissions has already entered the clinical trial stage in AML [28]. In the long term, it is likely that the introduction of MRD assessments will provide early end points in clinical trials and thus will modify the clinical landscape. However, to achieve this goal, standardization and harmonization processes of MRD detection methods and assays are required [28, 29]. Increasing the knowledge of the cellular MRD composition might also help in identifying relapse initiating cell types, which we will further discuss in the next section.

## AML biology and the cancer stem cell concept

AML is based on a differentiation defect of hematopoietic stem and progenitor cells (HSPCs) in the BM, resulting in accumulation of immature blast cells that displace the normal hematopoietic system. Within the BM microenvironment, AML blasts interact and communicate with stromal and immune cells, thereby impacting on the pathogenesis of the disease. In particular, leukemic blasts create their own protective niche by reprogramming mesenchymal stromal cells to selectively support leukemic cells, while simultaneously suppressing the normal hematopoiesis [30]. A common belief is that in AML a hierarchy of cells exists, with the most primitive types of cells residing in a quiescent state and protected in the leukemic niche representing the “leukemia initiating cells” or “cancer stem cells”. These cells are highly resistant to most chemotherapeutic drugs that mainly target cycling cells, and often give rise to MRD, which ultimately causes relapses [31, 32]. In a novel modification of this concept, the existence of pre-leukemic stem cells is also discussed [33, 34].

Stem cell pathways such as Wnt, Notch or Hedgehog (HH)/GLI signaling have been implicated in cellular self-renewal and resistance to chemotherapy of various cancer stem cell types [35].

The recent approval of small molecule inhibitors of HH/GLI signaling for the treatment of advanced and metastatic non-melanoma skin cancer has sparked high expectations that HH/GLI targeting may prove an efficient and even curative therapeutic approach for a range of solid and hematological malignancies [36–38]. However, several recent clinical trials have largely failed to demonstrate a therapeutic benefit of HH/GLI inhibitors that target the essential pathway effector Smoothened (SMO) in a variety of solid cancer entities [39]. These disappointing trial data dampened the enthusiasm of the field for treating HH-associated cancers by blocking SMO function but at the same time opened up new therapeutic strategies concentrating on the targeted inhibition of the critical oncogenic downstream HH effectors, the GLI zinc-finger transcription factors. There is substantial preclinical evidence that inhibition of SMO-independent GLI activation (henceforth referred to as non-canonical HH/GLI signaling) may provide a pronounced therapeutic benefit, also in settings with acquired or *a priori* resistance to SMO inhibitors [40–44].

In the following chapters, we aim to provide a concise overview of recent studies addressing the role of HH/GLI signaling in AML pathogenesis and its possible therapeutic implications. We summarize selected key mechanisms of non-canonical HH/GLI signal transduction, concentrating on novel insights into SMO-independent regulation of GLI activity by multiple oncogenic signal cues. Based on these cross-talk signaling events, we discuss possible therapeutic approaches tackling AML by targeting oncogenic GLI proteins with novel compounds and rational combination treatments.

## HH/GLI signaling in AML biology and therapy

**Fig. 1:**
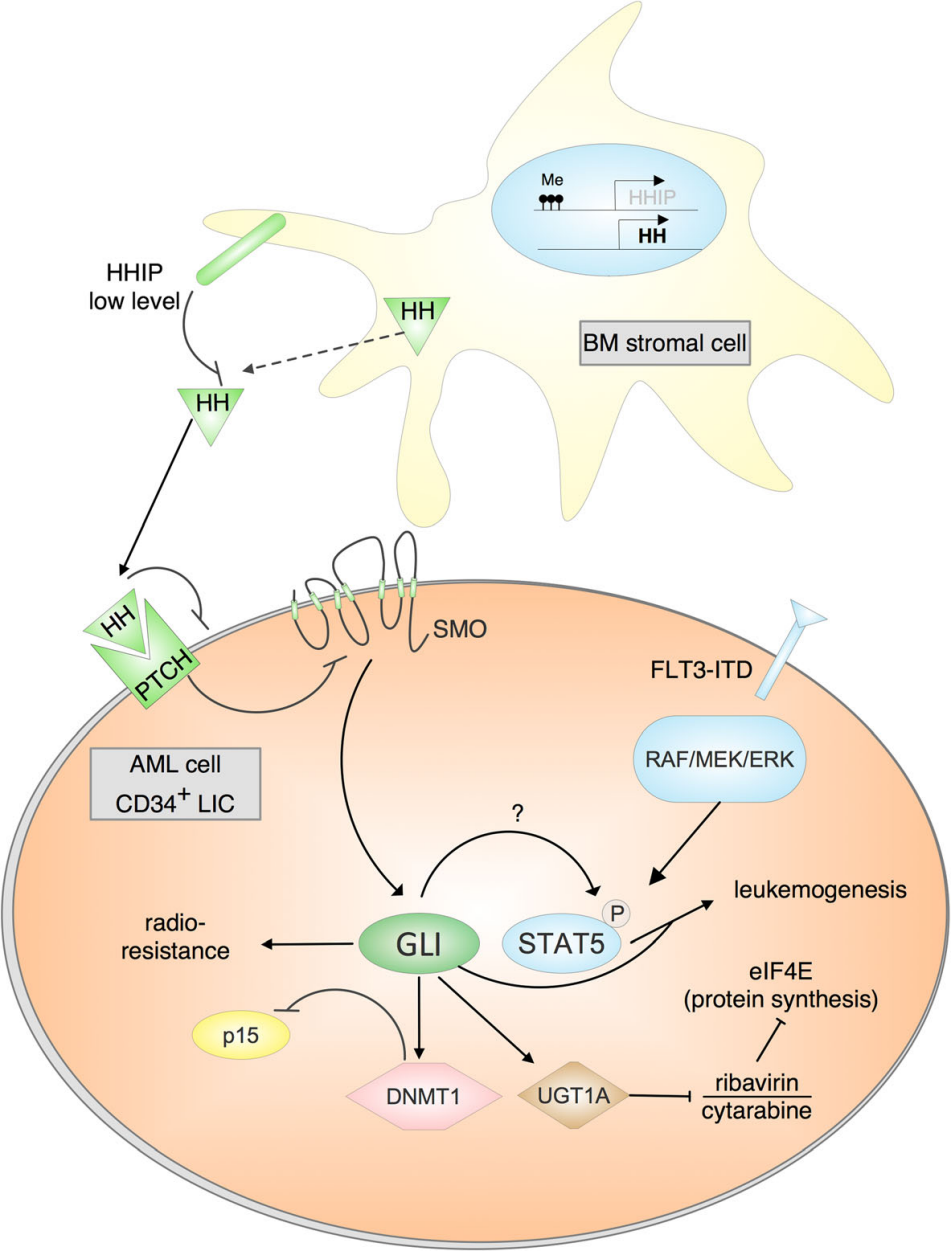
Model of oncogenic HH/GLI signaling in AML. Activation of HH/GLI in leukemic (stem) cells of AML patients can be activated by HH ligand derived from adjacent BM stromal cells expressing low levels of the HH inhibitor HHIP. GLI expression in AML cells can enhance radio- and chemoresistance, and promote leukemogenesis by epigenetically repressing cell-cycle inhibitors (e.g. p15) or by synergistic cross-talk with oncogenic FLT3/STAT5 signaling. LIC: leukemia initiating cell; Me: DNA methylation

With regard to AML biology and pathogenesis, the HH pathway has recently received much attention for its implication in leukemic stem cell regulation and in the orchestration of acquired drug resistance of poor prognostic AML (summarized in Fig. 1). Using modified human myeloid cell lines (HL60), Li and colleagues [45] showed that myeloid cells that acquired radio- (HL60/RX) as well as drug-resistance (HL60/ADR) express higher levels of SMO and GLI1. In line, the radioresistance was overcome by inhibition of the HH pathway via the SMO antagonist LDE225 (sonidegib/erismodegib) involving a cross-talk with and down-regulation of the GLI1/PI3K/AKT/NF-kB pathway. Thus, LDE225 treatment resulted in increased apoptosis induction and decreased DNA repair ability upon radiation.

Further evidence for an involvement of HH/GLI signaling in drug resistance was provided by Zahreddine et al. who analyzed primary tumor samples of patients that relapsed after monotherapy with ribavirin (an inhibitor of the eukaryotic translation initiation factor eIF4E) [46]. The authors observed an association of relapse and drug resistance with elevated levels of GLI1 and the UDP glucuronosyltransferase (UGT1A), which can inactivate ribavirin by glucuronidation, thus preventing binding of this drug to its target eIF4E. GLI alone was sufficient to drive the expression of UGT1A and accounted for drug glucuronidation. Accordingly, in vitro treatment of patient samples with previously failed induction therapy with the SMO inhibitor vismodegib (GDC-0449) potentiated the effects of cytarabine and ribavirin, providing a rationale for combination of HH inhibitors with conventional treatment regimes. Currently, a clinical trial using ribavirin and vismodegib with or without decitabine in AML is in the recruitment phase (clinical trial number NCT02073838). Patients with AML M4 or M5 FAB subtype or high eIF4E are eligible. All patients must have failed primary therapy (defined as two induction chemotherapies), must have relapsed, or must not be suitable candidates for intensive induction chemotherapy.

In addition, HH/GLI targeting also bears potential for those patients that do not tolerate aggressive therapeutic regimes. In particular, a combination of these antagonists with 5-Aza can be envisaged. Tibes and colleagues conducted an RNA interference sensitizer screen to identify gene targets of distinct regions presumably enhancing 5-Aza therapy [47]. Several HH pathway molecules could be identified, among them SMO, which was subsequently evaluated as a therapeutic target in vitro using seven heterogeneous AML cell lines. In these assays, the authors identified cytotoxic synergy of LDE225 and vismodegib with 5-Aza.

In fact, several clinical trials using SMO inhibitors alone or in combination with compounds blocking driver mechanisms in AML have already been initiated. For instance, the potency of the SMO inhibitor glasdegib (PF-04449913) alone or in combination with e.g. 5-Aza or chemotherapy is being investigated in several clinical trials for hematologic malignancies including MDS and AML (http://clinicaltrials.org, NCT01842646, NCT01841333, NCT01546038, NCT02367456). It is noteworthy that in a phase 2 trial with untreated AML and high-risk MDS patients, low dose Ara-C chemotherapy in combination with glasdegib improved overall survival when compared to chemotherapy only [48]. The community is keenly awaiting the outcome of these trials, also in light of the discussion of SMO-dependent and independent regulation of oncogenic GLI activity.

Indeed, besides targeting SMO, direct inhibition of GLI is a promising option, particularly in settings of SMO-independent GLI activation. On basis of two comprehensive clinical patient cohorts, a significant negative prognostic impact of GLI2 expression in AML could be established by Wellbrock and colleagues [49]. In the first cohort, based on the AMLSG 07–04 trial comprising 104 patient samples, the presence of GLI2 expression significantly shortened event-free survival, relapse-free survival, and overall survival and was correlated with FLT3 mutational status. Analysis of a second, independent cohort of 290 AML samples confirmed the negative impact of GLI2 on event-free survival and overall survival.

The relevance of GLI expression for disease pathogenesis was further strongly supported by in vitro and in vivo experimental data using treatment of AML cell lines by GANT61, a GLI antagonist tool compound [50], and GLI shRNA approaches as well as an adoptive transfer model of AML. Of note, mice transplanted with GLI1/GLI2 double-depleted AML cells displayed a moderate yet significant increase in survival compared to controls. These data clearly support the development of clinically useful GLI antagonists for therapy.

Further support for an association of FLT3-mutated AML and HH pathway activation via the STAT pathway has recently been provided by the Matsui group [51] using transgenic mouse models with a combination of constitutively active SMO and internal tandem duplications (ITD) of FLT3. Combined treatment using the SMO inhibitor saridegib/IPI-926 and the kinase inhibitor sorafenib resulted in reduced tumor load and increased survival of the mice. The clinical impact was further supported by the identification of increased GLI2 expression in FLT3-ITD positive AML patients.

In tumor cells, both autocrine and paracrine HH pathway activation has been described [52]. In AML, in contrast to some solid tumors, the microenvironment appears to play the dominant role in providing such activation inducing ligands to leukemic cells. Wellbrock and colleagues [49] observed that AML patients displayed increased serum levels of Desert Hedgehog (DHH) that was obviously produced and shed into the blood by the BM microenvironment rather than the AML cells. Similar observations have been made by other authors, reporting an increased expression of Sonic Hedgehog (SHH), SMO, and GLI1 in BM stromal cells of MDS patients, compared to healthy donors that are known to express Indian Hedgehog (IHH) and SMO [53], with even higher HH effector levels in post-MDS AML [54].

Human Hedgehog-interacting protein (HHIP), a glycoprotein binding to and thus inhibiting HH ligand function, is produced by healthy BM stromal cells and has been shown to have the potential of suppressing proliferation of leukemic cells. In contrast, HHIP expression in BM stromal cells derived from AML and MDS patients was reduced [55], which was accompanied by the ability of these cells to support leukemic cell proliferation. This reduced HHIP expression might thus contribute to the progression of AML and MDS. Moreover, pretreatment with 5-Aza induced demethylation of the *HHIP* gene and partial restoration of HHIP expression, thereby reducing the supportive effect of the primary AML/MDS stromal cells on the malignant cells and underlining the function of HHIP as an endogenous HH ligand inhibitor.

More recently, GLI1 expression has been shown to correlate with increased expression of DNA methyltransferase 1 (DNMT1) and high-risk MDS. Of note, knock-down of GLI1 in MDS cells not only decreased survival, proliferation and DNMT1 expression but also enhanced the demethylating efficacy of 5-Aza, resulting in lower methylation of the tumor suppressor gene p15 promoter and enhanced p15 expression, respectively [56]. Combined use of GLI antagonists with demethylating drugs may therefore show improved therapeutic efficacy.

In line with an oncogenic role of HH/GLI in MDS and AML, a positive correlation between GLI1 expression and percentage of AML blasts, as defined by CD34 expression, has recently been observed in BM [57]. Also in the cell lines used in this study, in vitro treatment with the GLI antagonist GANT61 resulted in reduced proliferative and colony forming characteristics and displayed synergistic cytotoxicity with Ara-C [57].

## The potential of targeting non-canonical HH/GLI signaling in AML

**Fig. 2:**
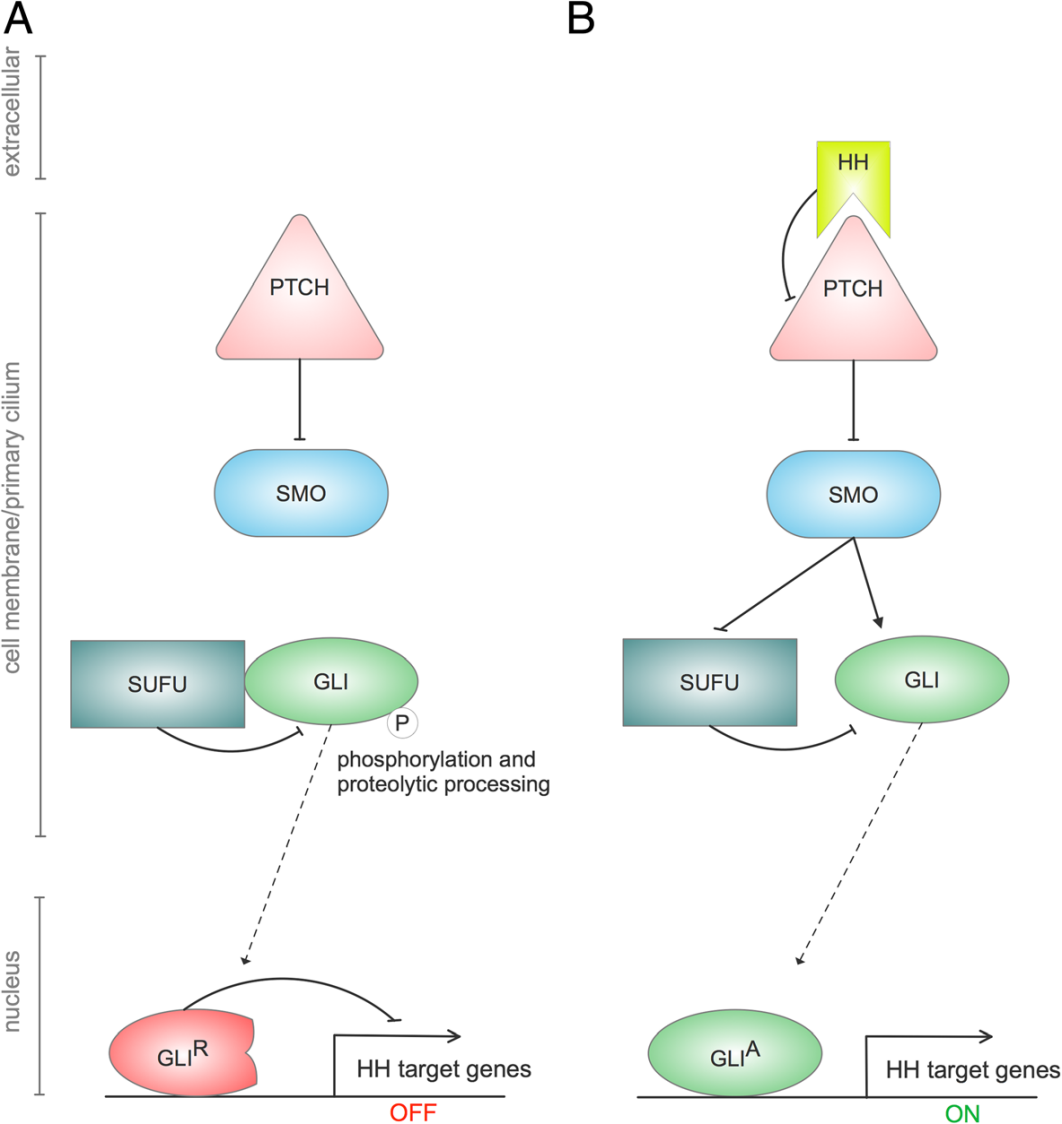
Canonical, ligand-dependent HH/GLI signaling. **a** In the absence of ligand, PTCH represses the ciliary translocation and activation of SMO, allowing the phosphorylation and proteolyic processing of full-length and SUFU-bound GLI protein into its C-terminally truncated repressor (GLI^R^) within the primary cilium. In the nucleus, GLI^R^ binds to promoters of direct HH-target genes to prevent their transcription. **b** Binding of processed and post-translationally modified HH protein to its receptor PTCH abolishes the inhibitory effect of PTCH on SMO, allowing ciliary transport and activation of SMO. Active SMO prevents GLI^R^ processing and induces release of active GLI from its repressor SUFU. GLI activator (GLI^A^) translocates to the nucleus, where it induces HH target gene expression

**Fig. 3:**
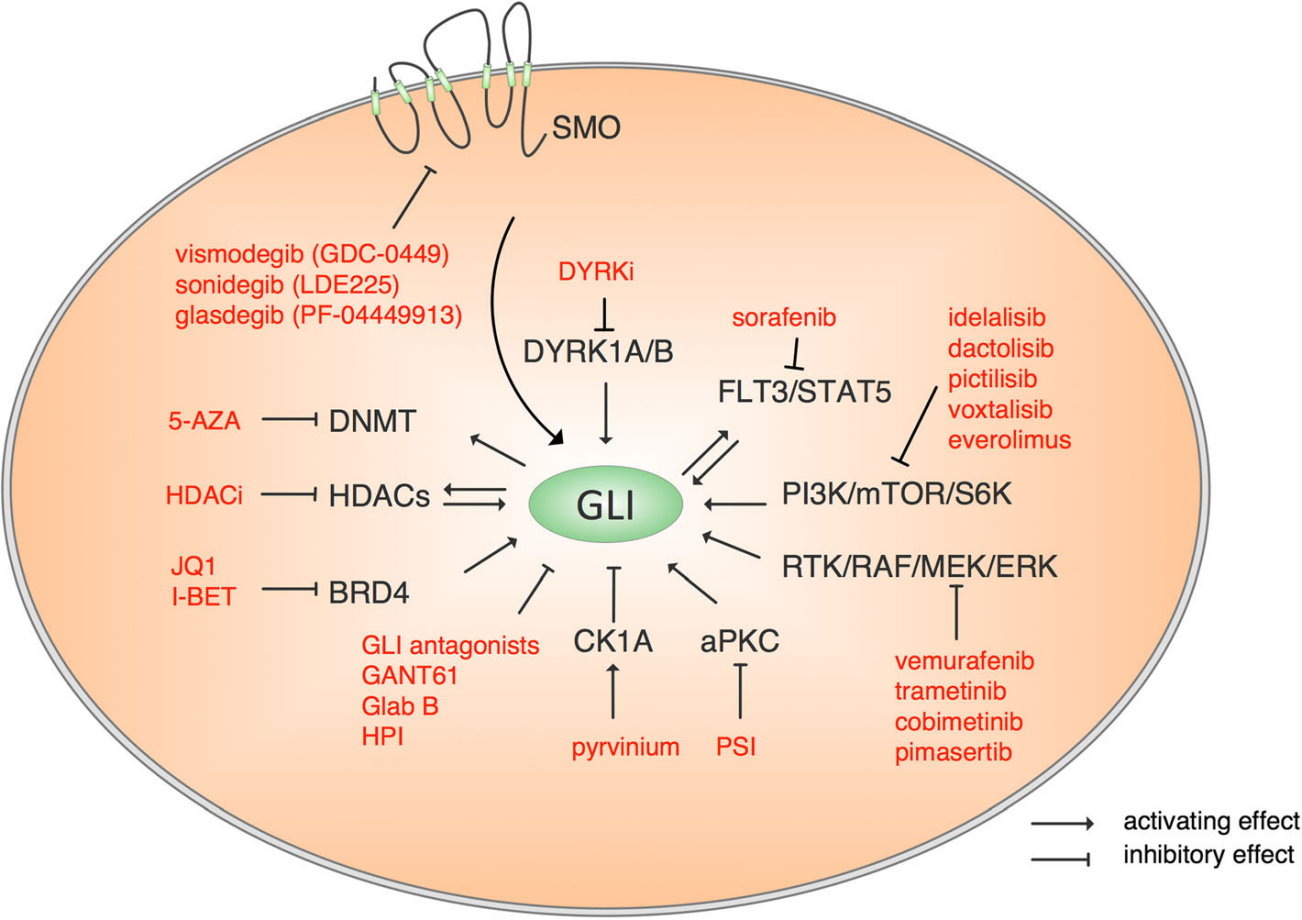
Non-canonical control of GLI activity by oncogenic mechanisms involving kinases, and epigenetic modulators. Rationale-based small-molecule targeting of these GLI regulatory signals with clinically approved/suitable drugs alone or in combination with HH pathway inhibitors such as direct GLI antagonists may generate efficient therapeutic effects. HPI-1: Hedgehog Pathway Inhibitor-1 acting at the level of GLI proteins [68], GANT61: direct GLI antagonist 61 [50], Glab B: glabrescione B (direct GLI inhibitor interfering with DNA binding) [67], HDACi: HDAC inhibitors

While canonical activation of the GLI transcription factors depends on activation of the essential pathway effector SMO either by ligand-dependent or genetic inactivation of the HH receptor Patched (PTCH) (Fig. 2) (reviewed in [58–61]), non-canonical regulation of GLI transcription factors is independent of SMO activation, and can be mediated by a variety of distinct key oncogenic signaling cascades [44, 62, 63]. This has important therapeutic implications, as SMO-independent GLI activation results in SMO inhibitor resistance, a clinically relevant problem that may account for the disappointing results from several trials using SMO inhibitors (e.g. vismodegib, sonidegib, saridegib) [39, 64]. Further, the severity of adverse effects induced by SMO inhibitors (e.g. muscle cramps) limits the prolonged administration of such drugs [65, 66]. We therefore propose that direct targeting of oncogenic GLI transcription factors, as has been shown in several proof of concept studies [50, 67, 68], in combination with strategies interfering with oncogenic cues promoting GLI activity in AML will provide a therapeutic benefit compared to single treatment protocols (Fig. 3).

In the following section, we will provide an overview of mechanistic models that can account for SMO inhibitor resistance and will summarize several representative and seminal studies that have revealed SMO-independent positive or negative regulation of GLI activity by oncogenic signaling pathways and epigenetic factors (reviewed in [44, 69]). Given the critical role of GLI transcription factors in the development and progression of several leukemic diseases including AML, a detailed understanding of “druggable” cross-talk pathways controlling oncogenic GLI function will provide the rationale for the design and evaluation of novel, efficacious combination treatments in AML.

## Oncogenic signals regulating GLI activity

The PI3K/AKT/mTOR and RAS/RAF/MEK/ERK signaling cascades control multiple cellular functions such as transcription, translation, proliferation, growth and survival. The aberrant activation of these oncogenic signals plays major roles in numerous malignant entities and therapeutic targeting of PI3K/AKT/mTOR and MEK/ERK signaling is a promising approach, intensely tested in clinical trials using selective small-molecule inhibitors (reviewed in [70–72]). Cross-talk of HH signaling with both PI3K/AKT and RAS/RAF/MEK pathways has been described in many cancer entities including melanoma, prostate cancer, non-melanoma skin cancer, glioma and leukemia. For instance, the cross-talk of HH/GLI and PI3K/AKT has an impact on GLI1 and GLI2 expression, protein stability, nuclear localization and transcriptional activity [73–80].

Another study dealing with human pancreatic cancer revealed an inhibitory effect on tumorigenic cancer stem cells through the combined blockade of HH and mTOR signaling using SMO and mTOR inhibitors together with standard chemotherapy [81]. These findings were supported by Miyazaki et al. who described that combined inhibition of HH and mTOR signaling eliminates pancreatic cancer stem cells. In contrast to the previously mentioned study, they used the direct GLI antagonist GANT61, which had a stronger negative effect on sphere formation and cell viability than the SMO inhibitor cyclopamine, even in the absence of additional standard chemotherapy [82].

Moreover, the signal integration of non-canonical GLI1/2 activation by PI3K/AKT was also deciphered as a novel potential therapeutic target because combination of the GLI inhibitor GANT61 and the AKT inhibitor perifosine resulted in synergistically suppressed tumor growth and induced apoptosis in renal cell carcinoma models [73]. Recently, Kern and colleagues reported a synergistic therapeutic effect in cells from a subgroup of CLL patients through combined targeting of GLI and PI3K/AKT/mTOR signaling [83]. Moreover, GLI1 protein can be phosphorylated by the ribosomal S6-kinase 1 (S6K1), a critical downstream effector of PI3K/AKT and MEK/ERK signaling. Notably, mTOR/S6K1-mediated phosphorylation appears to facilitate the release of GLI1 protein from its cytoplasmic repressor SUFU, thereby enhancing the overall oncogenicity of GLI1 in esophageal adenocarcinoma cells. In line with these mechanistic findings, combined inhibition of HH/GLI and mTOR/S6K1 activity synergistically reduced the survival of GLI expressing esophageal cancer cells [84]. Taken together, these studies support a pronounced therapeutic benefit of combined HH/PI3K/mTOR targeting in selected malignant diseases. Whether similar cooperative mechanisms operate in AML pathogenesis remains to be addressed.

GLI transcriptional activity is also positively regulated by RAS/RAF/MEK/ERK signaling, for instance in melanoma and pancreatic cancer [76, 85, 86]. Mechanistically, direct phosphorylation of GLI proteins by ERK kinases can enhance transcriptional GLI activity [86, 87]. Cross-talk of epidermal growth factor receptor (EGFR) signaling with HH/GLI also depends on MEK/ERK activation, yet involves another mechanism of cooperation. HH/EGFR signal integration relies on cooperativity of selected transcription factors simultaneously induced by concomitant HH/EGFR signaling. These studies revealed that EGFR can synergize with HH/GLI via MEK/ERK-dependent activation of JUN/AP-1 transcription factors, resulting in synergistic induction of common HH/EGF target genes and oncogenic transformation [78–80].

Additional druggable kinases modulating oncogenic GLI activity include atypical Protein Kinase C (aPKC or PKC iota/gamma) and members of the dual-specificity tyrosine phosphorylation regulated kinase (DYRK) family. aPKC has been shown to directly phosphorylate GLI1 at amino acid residues located in the zinc finger DNA binding domain, thereby enhancing DNA binding and maximum transcriptional activity of GLI. Notably, SMO inhibitor resistance can be mediated by hyperactivation of aPKC, suggesting that aPKC targeting in patients unresponsive to SMO inhibitors may overcome SMO inhibitor resistance, as shown in in vitro models [42, 88]. DYRK kinases can exert positive or negative effects on the transcriptional activity of GLI. DYRK1A-mediated phosphorylation of GLI1 can increase GLI1 activity by promoting its nuclear localization [89]. Recently, our own group has provided evidence for a critical positive role of DYRK1B rather than DYRK1A in various human cancer entities and shown that genetic and pharmacologic DYRK1B targeting can efficiently eliminate GLI1-dependent tumor-initiating pancreatic cancer cells [90]. Aside from this, a KRAS/DYRK1B network can also redirect autocrine HH signaling towards a paracrine mode in human pancreatic adenocarcinoma [91]. Unlike DYRK1 proteins, DYRK2 represses GLI activity by promoting proteasomal degradation of GLI2 via direct phosphorylation [92]. Pharmacologic inhibition of oncogenic GLI proteins therefore requires clinically useable drugs that selectively target DYRK1 family members.

Casein kinase 1-alpha (CK1A) and protein kinase A (PKA) can be considered additional potential therapeutic targets. Activation of CK1A by pyrvinium can promote GLI repressor formation and GLI degradation [93]. However, as CK1A can also be an activator of HH signaling, its overactivation has to be taken with precaution [94]. Activation of PKA for instance by imiquimod, a synthetic nucleoside analog that binds to adenosine receptors, induces GLI phosphorylation and subsequent degradation and/or cleavage into repressor forms [95].

In addition to phosphorylation, other post-translational modifications of GLI proteins control the oncogenic activity of GLI. Canettieri et al. have shown that acetylation of GLI1 and GLI2 represses, while histone deacetylase (HDAC)-mediated deacetylation increases their transcriptional activity. This interplay is further regulated by a positive feed-forward loop involving HH-induced upregulation of HDAC1 [96]. The role of HDACs in promoting HH/GLI signaling has been further supported by findings showing that HDAC6 activity is required for the full-activation of HH/GLI signal strength [97]. The use of selected clinically validated HDAC inhibitors, which have already shown promising therapeutic efficacy in AML patients [98], in combination with GLI antagonists may therefore be an attractive therapeutic approach in GLI-dependent cancer entities. The finding that a novel dual HDAC/SMO inhibitor, NL-103, can down-regulate both HH/GLI and HDAC activity, thereby overcoming vismodegib resistance [99], exemplifies that dual targeting of GLI and GLI promoting signals such as HDACs with a single compound is feasible and an attractive option for future therapeutic strategies including the treatment of AML patients.

As another epigenetic regulator of HH/GLI, the BET family member bromodomain 4 (BRD4) protein has been shown to modulate HH signaling. BRD4 can bind to acetylated lysines in histones, enhance target gene expression via stimulation of RNA polymerase II activity and can be efficiently inhibited by the BRD antagonists JQ1 and I-BET [100, 101]. BRD4 activity has been linked to HH/GLI signaling in two parallel studies showing that BRD4 regulates GLI transcription in a SMO- and SUFU-independent manner by binding directly to the GLI1 and GLI2 promoters and that JQ1 reduces the binding of BRD4 to its binding sites in the GLI promoters [101, 102]. Intriguingly, JQ1 also inhibits BRD4-regulated MYC activity [103], a critical driver signal in AML pathogenesis. The impressive therapeutic activity of BRD inhibitors in preclinical models of AML may therefore result from their inhibitory effect on multiple targets including key oncogenic players in leukemic (stem) cells such as MYC and GLI [104–107].

## Conclusions

In summary, the still very high occurrence of AML relapses upon therapy reflects the need for novel treatment strategies. In this regard, targeting the HH/GLI pathway in AML can be a promising therapeutic approach, since this signaling cascade is crucially involved not only in the regulation of cancer stem/leukemia initiating cells, but also in the development of drug resistance. The possibility of inhibiting multiple key players in this pathway (i.e. SMO, GLI1/GLI2) as well as the combination with other agents targeting important mechanisms involved in AML pathology (e.g. kinase inhibitors and epigenetic regulators such as 5-Aza, HDACi and BRD4) provide a multitude of new treatment options. Targeting the non-canonical HH/GLI signaling pathway by directly interfering with the activity of the GLI transcription factors as well as their cross-talk with other signaling pathways (e.g. kinases) may be particularly promising, since this alternate approach might prevent the development of resistance and severe side effects as seen for SMO inhibitors.

## Abbreviations

5-Aza: Azacitidine
AML: Acute myeloid leukemia
aPKC: Atypical Protein Kinase C
Ara-C: Cytarabine
BM: Bone marrow
BRD4: BET family member bromodomain 4
CK1A: Casein kinase 1-alpha
CR: Complete response/remission
DNMT1: DNA methyltransferase 1
DYRK: Dual-specificity tyrosine phosphorylation regulated kinase
ECOG: Eastern Cooperative Oncology Group
EGFR: Epidermal growth factor receptor
eIF4E: Eukaryotic translation initiation factor 4E
FAB: French-American-British
GLI: Glioma-associated Oncogene Homolog
HDAC: Histone deacetylase
HH: Hedgehog
HHIP: Hedgehog-interacting protein
HSC: Hematopoietic stem cell
HSPCs: Hematopoietic stem and progenitor cells
IHH: Indian Hedgehog
ITD: Internal tandem duplications
MDS: Myelodysplastic syndrome
MRD: Minimal residual disease
PKA: Protein kinase A
PTCH: Patched
rrAML: Relapsed/refractory AML
SHH: Sonic Hedgehog
SMO: Smoothened
UGT1A: UDP glucuronosyltransferase
WHO: World Health Organization
